# CD73 and AMPD3 deficiency enhance metabolic performance via erythrocyte ATP that decreases hemoglobin oxygen affinity

**DOI:** 10.1038/srep13147

**Published:** 2015-08-07

**Authors:** William G. O’Brien III, Vladimir Berka, Ah-Lim Tsai, Zhaoyang Zhao, Cheng Chi Lee

**Affiliations:** 1 Department of Biochemistry and Molecular Biology, University of Texas Health Science Center, Houston, Texas, USA 77030; 2 Division of Hematology, Department of Internal Medicine, University of Texas Health Science Center, Houston, Texas, USA 77030

## Abstract

Erythrocytes are the key target in 5′-AMP induced hypometabolism. To understand how regulation of endogenous erythrocyte AMP levels modulates systemic metabolism, we generated mice deficient in both CD73 and AMPD3, the key catabolic enzymes for extracellular and intra-erythrocyte AMP, respectively. Under physiological conditions, these mice displayed enhanced capacity for physical activity accompanied by significantly higher food and oxygen consumption, compared to wild type mice. Erythrocytes from *Ampd3*^−/−^ mice exhibited higher half-saturation pressure of oxygen (p50) and about 3-fold higher levels of ATP and ADP, while they maintained normal 2,3-bisphosphoglycerate (2,3-BPG), methemoglobin levels and intracellular pH. The affinity of mammalian hemoglobin for oxygen is thought to be regulated primarily by 2,3-BPG levels and pH (the Bohr effect). However, our results show that increased endogenous levels of ATP and ADP, but not AMP, directly increase the p50 value of hemoglobin. Additionally, the rise in erythrocyte p50 directly correlates with an enhanced capability of systemic metabolism.

We have previously reported that 5′-adenosine monophosphate (AMP) can safely induce mammals to enter a transient hypometabolism state that resembles the behavior of torpor observed naturally in many species of animals during prolonged periods of metabolic stress^1^. Our previous findings suggested that the uptake of AMP by erythrocytes alters intracellular adenine nucleotide ratios, leading to reduced oxygen transport capacity and induction of hypometabolism^2^,^3^. These findings prompted our further investigation into how erythrocyte AMP regulates oxygen transport by hemoglobin (Hb) in erythrocytes thus modulating systemic metabolism.

It is known that under physiological conditions, there are two main modulators of the erythrocyte’s gas transport function in mammals: 1) the reduction in pH due to dissolved CO_2_ levels (the Bohr effect) increases the release of oxygen in metabolically active tissues due to an increased Hb p50 in the lower pH micro-environment^4^; 2) the increase in a glycolytic metabolite, 2,3-bisphosphoglycerate (2,3-BPG), that directly binds to Hb, altering its quaternary structure leading to reduced affinity for oxygen^5^. The importance of 2,3-BPG in regulating the erythrocyte’s oxygen transport function was further supported by observations that erythrocytes from humans with a deficiency in 2,3-BPG mutase, the biosynthetic enzyme for 2,3-BPG, displayed reduced p50 values indicating an increased affinity for oxygen^6^. Many other organic phosphates, including adenine nucleotides were suggested to regulate Hb’s oxygen affinity^5^. However, it has been argued that the physiological concentrations of most of these organic phosphates were insufficient to have significant effects on Hb affinity for oxygen *in vivo*^7^. Further, erythrocytes lack *de novo* purine biosynthesis and their adenine nucleotide pools are thought to be relatively constant^8^.

We have previously proposed that oxygen transport was disrupted by AMP-uptake, which led us to ask whether these effects could be investigated by blocking AMP degradation in erythrocytes. Intracellular AMP is catabolized by two established pathways, dephosphorylation by cytosolic 5′-nucleotidase to adenosine, or deamination to IMP by AMP deaminase (AMPD)^9^. However, the K_m_ for adenosine deaminase is two orders of magnitude larger than the K_m_ of adenosine kinase for phosphorylating adenosine back to AMP^9^. Thus, the intracellular catabolism of AMP is primarily carried out by AMPD. Although there are three known AMPD isoforms, erythrocytes have only AMPD3^10^. To further validate the erythrocyte’s role in mediating 5′-AMP induced hypometabolism (AIHM), we generated mice deficient in AMPD3^3^. We reported that AMPD enzymatic activities were present in all tissues of *Ampd3*^−/−^ mice except the erythrocytes and that *Ampd3*^−/−^ mice were much more sensitive to AIHM, supporting our previous findings that erythrocytes are the cellular initiators of AIHM. Additionally, AMP is catabolized by CD73, the key extracellular ecto-nucleotidase responsible for dephosphorylating AMP to adenosine^11^. We also observed that the AIHM effect was more pronounced in the *Cd73*^−/−^ mice^2^. These findings led us to ask whether the loss of both CD73 and AMPD3 would lead to increased erythrocyte physiological adenine nucleotides levels, which could alter erythrocyte oxygen transport function.

In the current study, we generated mice deficient in both CD73 and AMPD3. Our studies revealed that these two regulators of AMP levels act synergistically to regulate systemic metabolism via regulation of adenine nucleotide (ATP, ADP and AMP) levels in erythrocytes, resulting in more significant changes in ATP and ADP levels than in those of AMP. We observed that an increase in endogenous concentrations of adenine nucleotides, particularly ATP, in AMPD3 deficient erythrocytes is sufficient to decrease Hb affinity for oxygen. This is reflected in corresponding increases in p50 values in *Ampd3*^−/−^ erythrocytes. The lower affinity for oxygen in erythrocytes increased the delivery of oxygen to tissues, especially working muscle, allowing *Ampd3*^−/−^/*Cd73*^−/−^ mice to maintain high metabolic activities over longer periods during wheel-running.

## Materials and Methods

### Mouse housing/husbandry/protocols

All animal studies were carried out in an approved animal facility by trained personnel under protocols HSC-AWC-13-012 and HSC-AWC-12-079 approved by Animal Welfare Committee (AWC), the institutional animal care and use committee (IACUC) at UTHSC-Houston. The *Ampd3*^−/−^/*Cd73*^−/−^ mouse line was generated by crossing *Ampd3*^−/−^ and *Cd73*^−/−^ mice previously described, back crossing the F1 mice, and selecting for *Ampd3*^−/−^/*Cd73*^−/−^ mice based on genotyping^3^,^12^.

### Wheel running experiments

Mice were placed in individual cages with a running wheel within a circadian chamber and provided with food and water ad libitum as previously described^13^,^14^. Briefly, wheel-running activity was measured continuously using Actiview Software. For each genotype both male and female mice (n = 4) of similar age were used. Mice were kept in a 12 h:12 h light:dark (LD) cycle for 2 weeks. The quantification of total wheel revolutions in zeitgeber time (ZT) was determined using Actiview software.

### Metabolic chamber experiments

A Comprehensive Lab Animal Monitoring System (CLAMS, Columbus Instruments, Columbus, OH) was used to measure animal metabolic rate as previously described^2^. Briefly, each chamber contained an individual mouse with free access to food and water. The O_2_ consumption and CO_2_ production of each mouse was monitored and recorded with the OxyMax System from Columbus Instruments. Each chamber has the option of wheel addition for running. The chamber was maintained at normal husbandry conditions under a 12 h:12 h LD cycle at 23 °C.

### Erythrocyte isolation

Each whole blood sample was collected from a clipped tail into a heparinized Eppendorf tube. The whole blood was then centrifuged at 4 °C at 3000 x g for one min. The plasma was removed, the erythrocytes were washed by resuspending cells in a 10X volume of ice-cold phosphate buffered saline (PBS) and centrifugation was repeated. After a total of three washes, the erythrocyte pellet was resuspended with a small volume of PBS. The cell count of the suspension was determined and it was then diluted with PBS to obtain 10^7^ cells/μl. The erythrocytes from all the genotypes were obtained and prepared in a similar manner.

### Blood and Hb oxygen saturation experiments

For all genotypes, blood oxygen saturation was measured by a Hemox Analyzer as previously described^15^. Solutions for the Hemox Analyzer were acquired from (TCS Scientific Corporation, New Hope, PA). Briefly, 10 μl of a 10^7^/μl suspension of erythrocytes was initially incubated at 37 °C in 4 ml of Hemox buffer (135 mM NaCl, 30 mM TES {N-[Tris (hydroxymethyl) methyl] -2- aminoethanesulfonic acid}, 5 mM KCl, pH 7.40 ± 0.02 at 37 °C; osmolarity 295 ± 10 mOsm/kg) and 0.1% BSA in the Hemox Analyzer After addition of 10 μl anti-foaming agent (AFA-25, TCS Scientific Corporation), pO_2_ and temperature were allowed to stabilize before measurement of oxygen saturation was undertaken. Hb Oxygen saturation was measured in a similar manner.

### 2,3-BPG Quantification

The quantification of erythrocytes 2,3-BPG was carried out using a kit (Roche Lifescience, Indianapolis, IN) based on methods previously described^16^. Briefly, erythrocytes were isolated, lysed and acid extracted, neutralized and processed following manufacturer’s instructions to quantify the amount of 2,3-BPG in the sample. In parallel, the protein concentration was measured using a Pierce BCA Assay kit (Thermo Fisher Scientific, Waltham, MA). The level of 2,3-BPG was then normalized to the level of total protein.

### Adenine nucleotide measurement

Adenine nucleotide measurements were carried out as previously described^17^. Briefly, a 2 X volume of 7.5% perchloric acid was added to isolated erythrocytes, mixed by vortexing and then centrifuged for 10 min at 4 °C at 13,000 x g. The supernatant was neutralized with equal parts of 0.75 M K_2_CO_3_, centrifuged at 13,000 x g for 10 min at 4 °C and the resulting supernatant was resolved on a HPLC C-18 column. The HPLC gradient buffers were as previously described^18^. Buffer A: 30 mM KH_2_PO_4_ + 0.8 mM tetrabutylammonium phosphate (TBAP) (#268100, Sigma, St Louis, MO) pH 5.45. Buffer B: an equal ratio (v/v) of acetonitrile to 30 mM KH_2_PO_4_, 0.8 mM TBAP, pH 7.0. After each run, a 20 min flush of the C-18 column was carried out with a buffer comprised of 90% Buffer A and 10% Buffer B. The amount of respective adenine nucleotide is quantified with AMP, ADP and ATP standards that were ran in parallel.

### Preparation of hemoglobin (Hb)

Stock solutions of 1 mM Hb (human Hb, #H7379, Sigma; mouse Hb, #324-30, Lee Biosolutions) were prepared by dissolving the lyophilized powder in 50 mM HEPES buffer, pH 7.4 and reduced with 10 mM of sodium hydrosulfite (#157953, Sigma) dissolved in 100 mM tetrasodium pyrophosphate buffer, pH 8.3 (#P8010, Sigma). Reduced Hb was applied to an Econo-Pac 10 DG Desalting column (Bio-Rad, Hercules, CA) that was pre-equilibrated with aerobic HEPES buffer to remove excess sodium hydrosulfite and to form Oxyhemoglobin (oxyHb). Electronic absorption spectra of oxyHb were recorded using a Hewlett-Packard 8452A diode-array spectrophotometer. The concentration of oxyHb was calculated using an extinction coefficient of 128 mM^−1^ cm^−1^ at 415 nm^19^.

### Methemoglobin (metHb) Measurement

Freshly isolated RBC were diluted in water (1:20). UV-VIS spectra of Hb from erythrocytes lysates were measured using a Hewlett-Packard spectrophotometer and a cuvette with a 2 mm path. The concentrations of oxyHb and metHb were calculated from absorption at 577 nm and 630 nm, respectively using extinction coefficients of 14.4 mM^−1^ cm^−1^ for oxyHb and 3.6 mM^−1^ cm^−1^ for metHb. The percentage of metHb in the samples was calculated as described previously^20^.

### Statistics

All data are plotted as average values +/− SEM with statistical analysis either by t-test or ANOVA, whichever was appropriate for the data set.

## Results

### Mice deficient in CD73 and AMPD3 display enhanced levels of locomotor and metabolic activities

**Figure 1:**
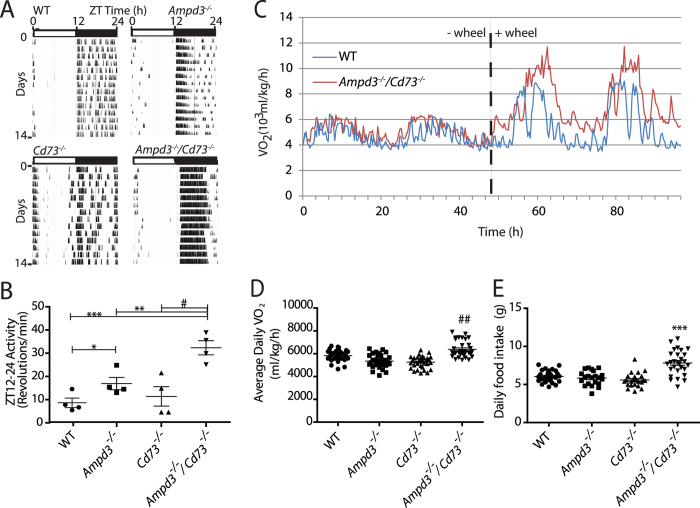
Loss of CD73 and AMPD3 alters locomotor and metabolic activities of mice. (**A**) Representative single plotted wheel running actograms of wild type, *Ampd3*^−/−^, *Cd73*^−/−^, and *Ampd3*^−/−^/*Cd73*^−/−^ mice in a 12:12h Light:Dark (LD) cycle. (**B**) The average wheel running activities between ZT12-ZT24 for the wild type, *Ampd3*^−/−^, *Cd73*^−/−^, and *Ampd3*^−/−^/*Cd73*^−/−^ mice (n = 4, each genotype). (**C**) Representative oxygen consumption (VO2) readings of a WT and *Ampd3*^−/−^/*Cd73*^−/−^mouse without and with access to an exercise wheel. (**D**) The daily average metabolic activity with access to a wheel for wild type, *Ampd3*^−/−^, *Cd73*^−/−^, and *Ampd3*^−/−^/*Cd73*^−/−^ (n = 4/genotype) mice over a period of seven days. (**E**) The daily food consumption by the mice in D. P values = * < .05, ^#^ < .01, ** < .005, ^##^ < .0005, and *** < .0001.

The *Ampd3*^−/−^/*Cd73*^−/−^ mice are fertile and morphologically indistinguishable from wild type mice. In an effort to find possible physiological abnormalities in *Ampd3*^−/−^/*Cd73*^−/−^ mice, we measured their locomotor activity assessed by wheel-running using age and sex matched wild type, *Ampd3*^−/−^*, Cd73*^−/−^ and *Ampd3*^−/−^/*Cd73*^−/−^ mice. Our wheel running analysis revealed that the *Ampd3*^−/−^/*Cd73*^−/−^ mice displayed enhanced levels of locomotor activity compared with wild type, *Ampd3*^−/−^ and *Cd73*^−/−^ cohorts (Fig. 1A,B). The actograms revealed that wheel running activities for *Ampd3*^−/−^/*Cd73*^−/−^ mice appeared largely continuous during ZT 12–24, unlike the other genotypes, whose periods of activity were interrupted by periods of rest. The wheel-running activity between ZT0-ZT12 was low and similar among the four genotypes, indicating that their activities are under circadian regulation. The average daily wheel running activity of an *Ampd3*^−/−^/*Cd73*^−/−^ mouse was about three fold higher than wild type. The average wheel running activity of an average *Ampd3*^−/−^ mouse was moderately but significantly elevated compared to wild type. Comparing *Ampd3*^−/−^/*Cd73*^−/−^ to *Ampd3*^−/−^ mice showed the latter had significantly less locomotor activity. Daily locomotor activity of an average *Cd73*^−/−^ mouse was not significantly different from wild type.

Next, we measured metabolic rate based on oxygen consumption (VO_2_) of mice in the presence and absence of running wheels. When running wheel access was blocked, we observed that the metabolic rates of *Ampd3*^−/−^/*Cd73*^−/−^ and wild type mice were comparable (Fig. 1C). When access to a running wheel was provided, both wild type and *Ampd3*^−/−^/*Cd73*^−/−^ mice displayed enhanced metabolic activities. However, the *Ampd3*^−/−^/*Cd73*^−/−^ mice exhibited significantly elevated average daily VO_2_ when compared to wild type (Fig. 1C,D). Providing access to a wheel for *Cd73*^−/−^ or *Ampd3*^−/−^ mice did not significantly enhance their average daily VO_2_ levels over wild type (Fig. 1D, Supplementary Fig. S1). We further undertook measurement of daily food consumption among the genotypes with access to wheel running. These data show that the *Ampd3*^−/−^/*Cd73*^−/−^ mice ate significantly more than *Ampd3*^−/−^*or Cd73*^−/−^ or wild type when access to wheel running was provided (Fig. 1E). Thus, the metabolic activities of mice appear to correlate in part with food intake. No significant difference in blood glucose levels during fasting was observed among mice of the four genotypes suggesting that the ability to maintain glucose homeostasis does not appear to be significantly affected by the enzyme deficiencies (supplementary Fig. S2). Together, these observations suggest that loss of both AMPD3 and CD73 functions have additive effect on the capacity for metabolic activity and food consumption in mice, compared to mice deficient in only one of these genes.

### Loss of AMPD3 alters erythrocyte oxygen saturation level

**Figure 2:**
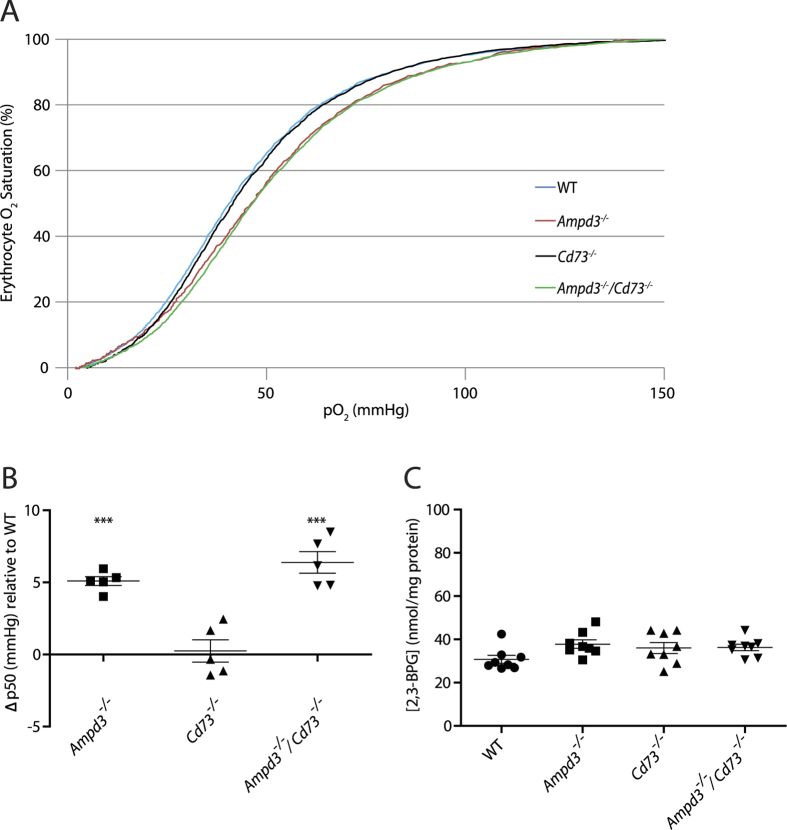
Erythrocytes’ p50 is increased in mice deficient in AMPD3. (**A**) Representative profiles of oxygen saturation vs oxygen pressure of isolated erythrocytes from WT, *Ampd3*^−/−^, *Cd73*^−/−^, and *Ampd3*^−/−^/*Cd73*^−/−^ mice. (**B**) Quantitative change in isolated erythrocytes from *Ampd3*^−/−^*, Cd73*^−/−^, and *Ampd3*^−/−^/*Cd73*^−/−^ p50 value with respect to WT erythrocytes. (**C**) Erythrocytes 2,3-BPG levels in WT, *Ampd3*^−/−^, *Cd73*^−/−^, and *Ampd3*^−/−^/*Cd73*^−/−^ mice. Each plotted point is an individual mouse. P values = *** < .0001.

We reasoned that the observed prolonged metabolic activity phenotype of *Ampd3*^−/−^/*Cd73*^−/−^ mice could be linked to changes in the erythrocytes main function of oxygen transport. Thus, using a Hemox analyzer we measured the pressure required for 50% oxygen saturation (p50) in isolated erythrocytes from wild type, *Ampd3*^−/−^*, Cd73*^−/−^ and *Ampd3*^−/−^/*Cd73*^−/−^ mice. Strikingly, the p50 value was significantly increased in *Ampd3*^−/−^ erythrocytes, by 4 to 6 mmHg (Fig. 2A,B), when compared to erythrocytes from wild type. Relative to wild type, the p50 was also increased in *Ampd3*^−/−^/*Cd73*^−/−^ erythrocytes by a range of 5 to 9 mmHg. The p50 values obtained for erythrocytes from *Cd73*^−/−^ were not significantly different from wild type. The right shift of the p50 curve indicates that a higher pressure is necessary for the erythrocytes to reach 50% oxygen saturation. This indicates that the cell’s affinity for oxygen is reduced, facilitating the release of oxygen from the erythrocyte to tissues.

Since 2,3-BPG is known as the main intracellular modulator of Hb’s affinity for oxygen and an increase in its concentration shifts the Hb oxygen dissociation curve rightward, we measured 2,3-BPG levels in erythrocytes from all four genotypes. Measurements revealed no significant difference in 2,3-BPG levels among the erythrocytes of the different genotypes (Fig. 2C). Thus, these findings indicate that the increase in p50 value is not due to a rise in intracellular 2,3-BPG levels.

Erythrocytes from each of the genotypes all showed a pH of 7.4 in Hemox reaction medium, consistent with the medium pH. To measure erythrocyte intracellular pH, 100 μl samples of isolated erythrocytes from each genotype were lysed in 0.5 ml of deionized water, then the pH of these diluted cellular lysates was measured. The erythrocyte’s intracellular pH value was 7.2 for wildtype, and 7.3 for *Ampd3*^−/−^, *Cd73*^−/−^ and *Ampd3*^−/−^/*Cd73*^−/−^ mice. The values measured were consistent with the physiological pH range of the erythrocyte’s cytosol, between pH 7.1–7.3^21^. The p50 measurements were carried out at 37 °C, and thus the effects of temperature on Hb affinity for oxygen could be excluded. The methemoglobin (metHb) levels were measured in the erythrocytes of the four genotypes and obtained values (<5%) that were comparable among genotypes. (Supplementary Fig. S3). Together, we have observed that mice deficient in AMPD3 have an enhanced erythrocyte p50 value that is not linked to an increase in intracellular metHb, 2,3-BPG levels or a change in intracellular pH or temperature.

### Elevated adenine nucleotide levels in AMPD3 deficient erythrocytes

**Figure 3:**
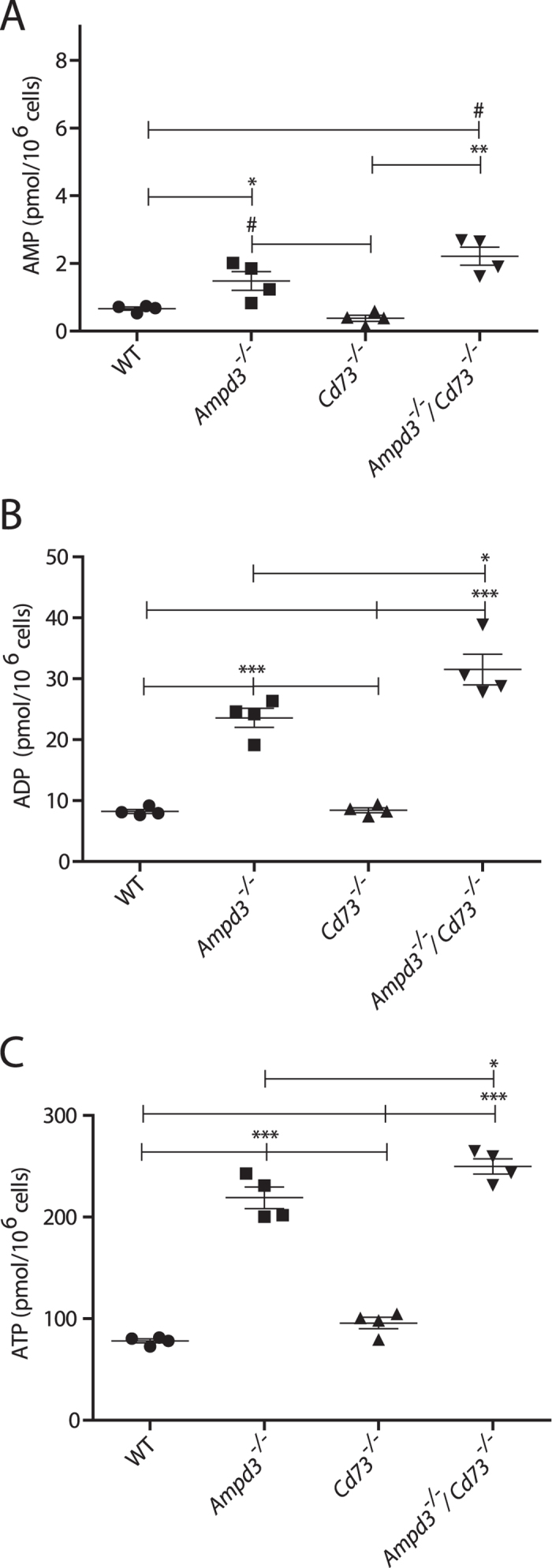
Erythrocytes’ adenine nucleotide levels are increased in mice deficient in AMPD3. WT, *Ampd3*^−/−^, *Cd73*^−/−^, and *Ampd3*^−/−^/*Cd73*^−/−^ mice erythrocyte levels of: (**A**) 5′-AMP, (**B**) ADP and (**C**) ATP. Each plotted point is an individual mouse. P values = * < .05, ^#^ < .01, ** < .005, and *** < .0001.

We reasoned that in *Ampd3*^−/−^/*Cd73*^−/−^ mice, blockade of AMP catabolism in erythrocytes could alter the levels of ATP and ADP, as well as AMP, since ATP and ADP must first be dephosphorylated to AMP before they can be further catabolized^22^. Utilizing HPLC, we measured the levels of adenine nucleotides after perchloric acid extraction of erythrocytes from the four genotypes (Fig. 3A–C). Obtained data showed that total adenine nucleotides were significantly increased in *Ampd3*^−/−^ erythrocytes largely due to increases in ATP and ADP rather than AMP. AMP levels in *Ampd3*^−/−^ erythrocytes are about two fold those of *Ampd3*^+^/^+^ erythrocytes. By contrast, ADP and ATP levels were about three fold increased in *Ampd3*^−/−^ erythrocytes compared with *Ampd3*^+^/^+^ erythrocytes. Consistent with previous reports and irrespective of the mouse genotype, we observed that the erythrocytes ratios of [ATP] to [ADP] and [ATP] to [AMP] were maintained at approximately 10:1 and 100:1, respectively^5^,^23^.

Thus, ATP comprises the majority of the adenine nucleotide pool. Comparison of *Ampd3*^−/−^/*Cd73*^−/−^ to *Ampd3*^−/−^ erythrocytes showed that ATP and ADP but not AMP were significantly elevated. Relative to wild type erythrocytes, the ATP levels of *Ampd3*^−/−^/*Cd73*^−/−^ and *Ampd3*^−/−^ erythrocytes showed an average increase of 3.2 and 2.8-fold, respectively (Fig. 3B,C). These findings suggest that AMPD3 is the major regulator of the erythrocyte adenine nucleotide pool, while CD73 plays a lesser but significant role.

Interestingly, the different adenine nucleotides levels in erythrocytes among the four genotypes correlate well with their erythrocyte p50 values. This correlation suggests that the observed increase in *Ampd3*^−/−^ erythrocyte’s p50 values could be linked to their increased cellular adenine nucleotide levels.

### Modulation of hemoglobin’s oxygen saturation by ATP

Previous studies have demonstrated that many organic phosphates can bind Hb and modulate its affinity for oxygen^5^. However, it has been proposed that except for 2,3-BPG, the majority of these organic phosphates are present at physiological concentrations that are too low to be effective moderators of Hb affinity for oxygen *in vivo*^7^.

In *Ampd3*^−/−^/*Cd73*^−/−^ mice, the endogenous erythrocyte adenine nucleotides are several fold higher than wild type. Therefore, we investigated whether these increased levels of adenine nucleotides are sufficient to alter Hb’s affinity for oxygen and account for the increase in p50 values. The physiological intracellular concentration of ATP in wild type erythrocytes is estimated to be between 1–2.5 mM^5^,^24^, ADP 0.1–0.25 mM and AMP 0.01–0.02 mM^5^. To measure the effect of adenine nucleotides on p50 values we titrated purified human adult Hb (Hb-A) with different concentrations of ATP, ADP, and AMP in the presence of 1 mM 2,3-BPG.

**Figure 4:**
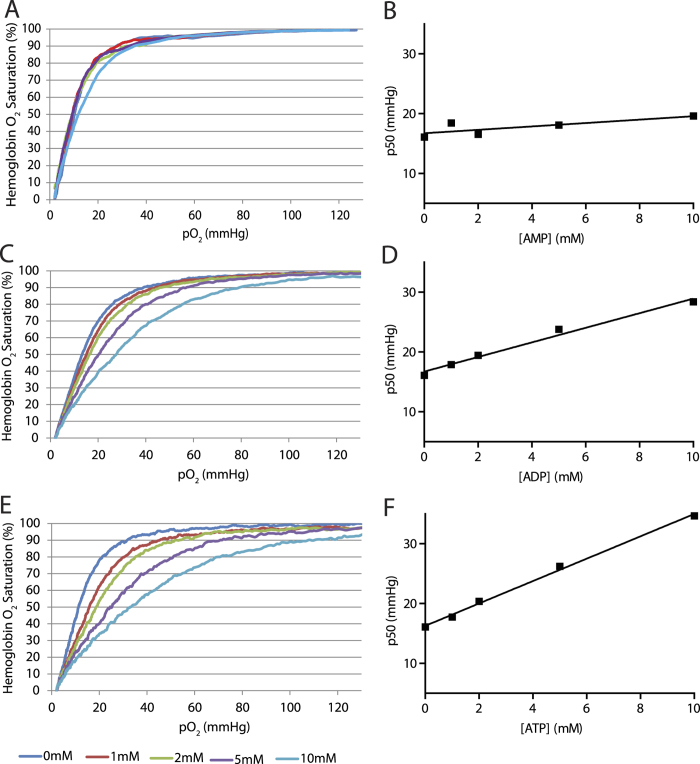
Adenine nucleotides’ effects on p50 of hemoglobin. Adenine nucleotide titrations of human hemoglobin p50 (200 μM) in the presence of 1mM 2,3-BPG: (**A**,**B**) 5′-AMP, (**C**,**D**) ADP, and (**E**,**F**) ATP.

We observed that the Hb-A p50 value is only weakly affected by AMP (Fig. 4A,B). Even at 10 mM AMP the increase in Hb-A p50 value was about 1 mmHg. Thus, the effect of AMP on Hb’s p50 value is weak and the observed amount of AMP increase in AMPD3-deficient erythrocytes could not have altered its p50 value significantly.

The effect of ADP on Hb-A p50 value was more prominent (Fig. 4C,D). There was a linear correlation between the rise in ADP concentration from 0 mM to 10 mM and the rise in Hb-A p50 value. Based on Figs 4D and 3B, we estimated a 3-fold rise in ADP concentration in *Ampd3*^−/−^ erythrocytes would contribute about a 0.2–0.5 mmHg rise in the p50 value of Hb-A. Therefore, the increase in intracellular ADP is likely a small contributor to the observed rise in *Ampd3*^−/−^ erythrocytes’ p50 value.

Next we titrated the ATP concentration against the Hb-A p50 value. Similar to ADP, increasing ATP concentrations from 0 mM to 10 mM caused a linear rise in the p50 values of Hb-A (Fig. 4E,F). The rate of change of the p50 value as a function of nucleotide concentration is much greater for ATP (slope m = 1.87) than for ADP (m = 1.22) indicating that ATP is more effective as a modulator of Hb-A p50 value. We observed that the effects of the respective adenine nucleotides on purified mouse Hb p50 were similar to those of human Hb-A (supplementary Fig. S4). Based on physiological concentrations of ATP in wild type mammalian erythrocytes of 1.0–2.5 mM, using data from Fig. 3C,F, we estimate a 3 fold increase in ATP concentration in *Ampd3*^−/−^ versus wild type erythrocytes would generate an increase in Hb p50 value of 4–9 mmHg. This value is consistent with their observed average difference in erythrocyte p50 values of about 6 mmHg. These findings suggest that the shift in p50 is primarily caused by the increase in intracellular ATP levels in the *Ampd3*^−/−^ erythrocytes.

## Discussion

Energy homeostasis in a living organism is maintained by regulation of systemic metabolism. The role of erythrocytes in regulation of systemic metabolic control is well recognized, as illustrated by artificially boosting the number of erythrocytes in circulation, widely known as blood doping, to enhance athletic performance^25^,^26^, or by the induction of hypothermia through blood loss by exsanguination^27^,^28^. In our previous studies we have proposed that the adenylate equilibrium was perturbed upon an acute uptake of AMP by erythrocytes leading to systemic metabolic repression^23^. These observations led us to pose the question of how erythrocyte adenine nucleotide levels regulate its function in oxygen transport and, thereby, systemic metabolism. Since ATP is the currency of consumable energy for biochemical reactions in cellular processes, its production and consumption must be tightly regulated. The energy charge of the adenine nucleotide pool, as reflected in the adenylate equilibrium, ATP + AMP ¤ 2ADP, results from the balance of energy production and consumption in a cell. The ultimate availability of ATP depends on the level of the adenine nucleotide pool, the sum of ATP, ADP, and AMP. The size of the adenine nucleotide pool depends on the synthesis and degradation of one component of this pool, AMP^22^. The intracellular catabolism of AMP is primarily carried out by AMPD. The three known tissue-specific isoforms of the enzyme, AMPD1, AMPD2 and AMPD3, are expressed in muscle, liver, and erythrocytes, respectively^10^. AMPD3 is the only isoform present in erythrocytes and is expressed in other tissues at low levels. *Ampd3* expression is activated in response to energy imbalance^29^, oxidative stress^30^ and Ca^2+^-calmodulin signaling^31^. Unlike cells of other tissues, erythrocytes have no salvage pathway that converts IMP back to AMP, since they are deficient in adenylosuccinate synthetase^32^. Thus, catabolism of AMP by AMPD3 in erythrocytes results in an irreversible loss of this nucleotide from the adenine nucleotide pool. Therefore, blocking AMP’s main catabolic pathway by targeted gene disruption of *Ampd3* could result in an enlarged pool of adenine nucleotides. The cellular adenylate equilibrium preferentially maintains the majority of the adenine nucleotide pool as ATP. Indeed, humans lacking AMPD3 have a large increase in ATP levels in their erythrocytes^33^ as also observed in mice deficient in AMPD3^3^,^34^.

We and others have observed that plasma AMP is readily taken up by erythrocytes^2^,^35^. In order to further understand how erythrocyte AMP levels regulate systemic metabolism, we generated mice deficient in both intra- and extracellular AMP catabolic enzymes, the *Ampd3*^−/−^/*Cd73*^−/−^ model. Unexpectedly, we observed that the level of wheel running by *Ampd3*^−/−^/*Cd73*^−/−^ mice was on average 3 fold higher than wild type and *Cd73*^−/−^ mice and about 1.5 fold higher than *Ampd3*^−/−^ mice. This differential in wheel running was supported by their elevated daily VO_2_ and food intake compared to those of wild type, *Cd73*^−/−^, or *Ampd3*^−/−^ mice. These observations suggest that the metabolic regulatory pathway of extracellular CD73 and intra-erythrocyte AMPD3 is physiologically connected. Interestingly, in the absence of work (wheel-running), the basal metabolic activities among the four genotypes were comparable. This suggests that much of the increase in VO_2_ of the *Ampd3*^−/−^/*Cd73*^−/−^ mice was driven by increased muscle activity during work (wheel-running) and not a general increase in basal cellular metabolic state. We reasoned that erythrocytes of the *Ampd3*^−/−^/*Cd73*^−/−^ mice likely were able to deliver greater levels of oxygen to working muscles thereby allowing for sustained levels of wheel running activity leading to greater levels of oxygen consumed. Consequently, to replenish the larger energy expenditure during work, the *Ampd3*^−/−^/*Cd73*^−/−^ mice on average ate more food.

This raised the question of how the loss of both CD73 and AMPD3 results in erythrocytes delivering greater amounts of oxygen to muscle during wheel running. We reasoned that this synergy stems from the fact that together they regulate the erythrocyte adenine nucleotide pool, acting at different cellular locations. When erythrocyte adenine nucleotides were measured in the four genotypes, the total levels of adenine nucleotides in AMPD3 deficient mice (both *AMPD3*^−/−^ and *Ampd3*^−/−^/*Cd73*^−/−^) were about three fold higher than those from wild type or *Cd73*^−/−^ mice. Remarkably, the ratio of ATP: ADP: AMP is still maintained close to 100:10:1 by the adenylate equilibrium in the *Ampd3*^−/−^ erythrocytes. Consequently, the majority of this increase in adenine nucleotides came from the increase in ATP levels. When comparing the levels of ATP and ADP in *Ampd3*^−/−^/*Cd73*^−/−^ versus *Ampd3*^−/−^ erythrocytes, the levels in the former were significantly elevated suggesting CD73 also contributes to the increase of intracellular levels of adenine nucleotides in erythrocytes.

These findings lead to the question of how the change in erythrocyte adenine nucleotide levels results in an increase in systemic metabolism. We reasoned that erythrocytes’ regulation of metabolism likely involves regulation of its main biological function of oxygen transport. When comparing the four genotypes, the p50 values of erythrocytes that were deficient in AMPD3 were on average about 6 mmHg higher compared with those of AMPD3 proficient cells, indicating that the loss of AMPD3 is the main contributor to the observed increase in the p50 values. Intriguingly, while the average p50 value of *Ampd3*^−/−^/*Cd73*^−/−^ erythrocytes is noticeably higher than that from the *Ampd3*^−/−^ genotype, the p50 values of CD73^−/−^ erythrocytes were similar to that of wild type. These observations suggest that the role of CD73 in regulating erythrocyte adenine nucleotides becomes more prominent in the absence of AMPD3.

We then examined how changes in adenine nucleotide levels modulate the p50 of Hb. Do they act as direct modulators or do they act via changes in 2,3-BPG, metHb or pH? We found erythrocyte 2,3-BPG levels among the four genotypes were similar. Furthermore, intracellular pH values of all 4 genotypes were well within the established pH range of normal erythrocytes^21^. Furthermore, we observed that a change in extracellular pH affects p50 values proportionately among the erythrocytes of the four genotypes (supplementary Fig. S5). We also examined a possible effect of metHb. There was no significant difference between erythrocytes from mice of all four genotypes. Based on these findings, we conclude that the increase in p50 values observed in *Ampd3*^−/−^ erythrocytes was not driven by increased 2,3-BPG levels , decreased intracellular pH or a rise in metHb levels. We then asked whether adenine nucleotides can regulate the p50 of Hb either directly or through another mechanism. Studies have shown Hb’s affinity for oxygen can be regulated by many types of organic phosphates^5^. These observations led us to examine the effects of adenine nucleotides on the p50 values of purified Hb-A. Consistent with previous findings, we observed that ATP and ADP increased purified Hb-A p50 values. We observed a linear correlation between increasing concentrations of ATP or ADP with increases in p50 values of Hb-A. Consistent with previous findings, we observed that AMP has only a minimal effect on purified Hb-A affinity for oxygen^36^. While ADP can significantly alter Hb affinity for oxygen and increase its p50 value, the concentration required to shift the p50 by about 6 mmHg was estimated to be about 5 mM based on the correlation between p50 values and ADP concentration shown in Fig. 4D. Given that the physiological concentration of intracellular ADP in wild type erythrocytes is between 0.1–0.2 mM, a three-fold increase in this concentration cannot account for the observed *Ampd3*^−/−^ erythrocytes’ increase in p50 values. The concentration of ATP in wild type erythrocytes is between 1–2.5 mM^5^,^24^. We observed ATP at this concentration can potently increase Hb’s p50 values. Our present study shows that the loss of AMPD3 results in about a three-fold increase in ATP levels compared with wild type erythrocytes. This would put the estimated ATP concentration in AMPD3 deficient erythrocytes at between 3–7.5 mM, similar to the 2.55 mM K_d_ of ATP binding to Hb-A^37^. Based on the correlation between ATP concentration and the increase in Hb p50 value, we estimate this increase in *Ampd3*^−/−^ erythrocyte ATP levels would generate an increase of between 4 to 9 mmHg in Hb p50 values, in line with the increase observed in *Ampd3*^−/−^ erythrocytes. Thus, it is reasonable to conclude that accumulation of ATP in erythrocytes contributes to the majority of the increase in p50 values of AMPD3-deficient over normal erythrocytes. Our studies identify AMPD3 as a major regulator of the adenine nucleotide pool in the erythrocytes. While the loss of CD73 itself appears to have little impact on the erythrocytes’ p50 value, we can only speculate on the impact of extracellular AMP influx into erythrocytes deficient in AMPD3 *in vivo*. We reason that AMP influx must exert a dynamic and significant impact on the erythrocytes’ ability to release oxygen since systemic metabolism and the total adenine nucleotide pool are significantly elevated in *Ampd3*^−/−^/*Cd73*^−/−^ compared to *Ampd3*^−/−^ mice.

It has been argued that Hb binding of oxygen is too tight in the absence of organic phosphate to be physiologically useful^38^. Hb binding of 2,3-BPG lowers the Hb affinity for oxygen to a range where H^+^, CO_2_ and other organic phosphates that occur at lower physiological concentrations could then fine-tune Hb affinity for oxygen. Since 2,3-BPG is a metabolite that can be synthesized without perturbing the adenylate ratios, the possible role of the adenine nucleotides in modulating Hb affinity for oxygen has not been considered important^38^. By contrast, other studies have implicated the importance of ATP in regulating Hb affinity for oxygen^39^. In some ectothermic species, such as fish, ATP has been shown to be a major mediator of Hb’s affinity for oxygen^40^. Our present studies indicate that the mammalian erythrocyte ATP level is a major modulator of Hb affinity for oxygen, as also the case in ectothermic species. Previous studies have demonstrated that both ATP and 2,3-BPG bind to Hb with comparable affinity^37^,^39^,^41^,^42^. Here, we have carried out measurements of ATP effects on Hb-A p50 values in the absence and presence of 2,3-BPG (supplementary Fig. S6). The p50 values obtained in the presence and absence of 2,3-BPG over a range of ATP concentrations were nearly parallel suggesting that 2,3-BPG and ATP effects on Hb-A p50 values are additive. Since both of these organic phosphates occur in erythrocytes at physiological concentrations in the millimolar range, it is likely that the Hb p50 value is regulated in part by the total intracellular levels of 2,3-BPG and ATP. Thus, we propose that a change in erythrocyte ATP levels during glycolytic and metabolic activities could be a mechanism to achieve finer dynamic control of oxygen delivery to tissues.

In conclusion, our current studies suggest that regulation of erythrocytes’ affinity for oxygen could regulate systemic metabolism. We show that increased adenine nucleotide levels, ATP in particular, correlate with increased erythrocyte p50 values. We demonstrate using purified Hb *in vitro* that physiological ATP and ADP concentrations can directly modulate Hb p50 at a value consistent with observed erythrocyte p50 values *in vivo*. A rise in ATP directly leads to increased Hb p50 values in turn leading to erythrocytes’ reduced affinity for oxygen. This decrease in affinity results in release of oxygen at a faster rate to working muscle and other tissues accounting for the enhanced metabolism observed in *Ampd3*^−/−^/*Cd73*^−/−^ mice during wheel running.

## Additional Information

**How to cite this article**: O’Brien, W. G. *et al.* CD73 and AMPD3 deficiency enhance metabolic performance via erythrocyte ATP that decreases hemoglobin oxygen affinity. *Sci. Rep.* **5**, 13147; doi: 10.1038/srep13147 (2015).

## Supplementary Material

Supplementary Information
