# Supplementary material for: CD73 and AMPD3 deficiency enhance metabolic performance via erythrocyte ATP that decreases hemoglobin oxygen affinity
Source: Sci Rep. 2015 Aug 7;5:13147. doi: 10.1038/srep13147 (PMC4650700; doi:10.1038/srep13147)
Supplement: Supplementary Information [file srep13147-s1.pdf]

Supplementary Figures for

CD73 and AMPD3 deficiency enhance metabolic performance via erythrocyte ATP that decreases hemoglobin oxygen affinity

William G O'Brien[1], III, Vladimir Berka[2], Ah-Lim Tsai[2], Zhaoyang Zhao[1] and Cheng Chi Lee[1*]

[1]Department of Biochemistry and Molecular Biology, University of Texas Health Science Center, Houston, Texas, USA 77030.

[2]Division of Hematology, Department of Internal Medicine, University of Texas

Health Science Center, Houston, Texas, USA 77030.

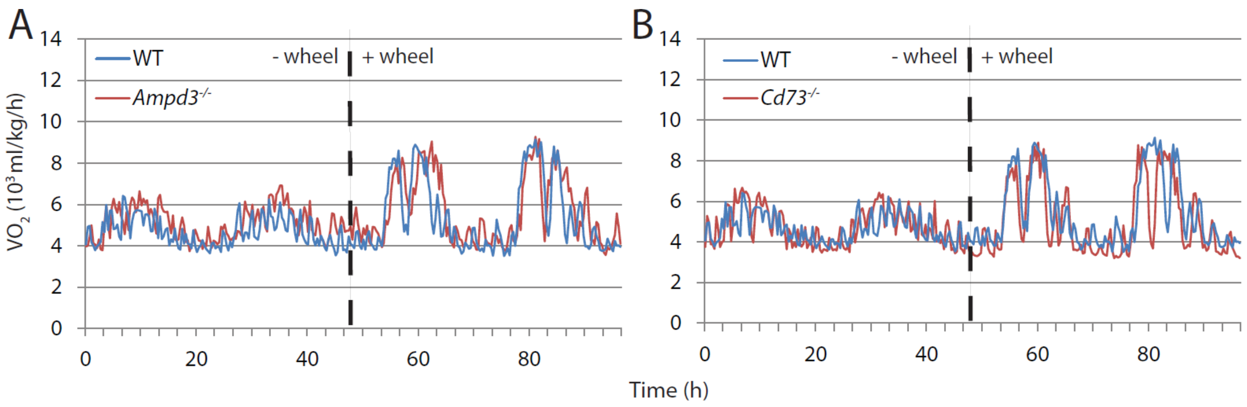

Figure S1, Comparing oxygen consumption of WT mice to *Ampd3*$^{-/-}$ (A) and *Cd73*$^{-/-}$(B) with and without running wheel over periods of 48 h.

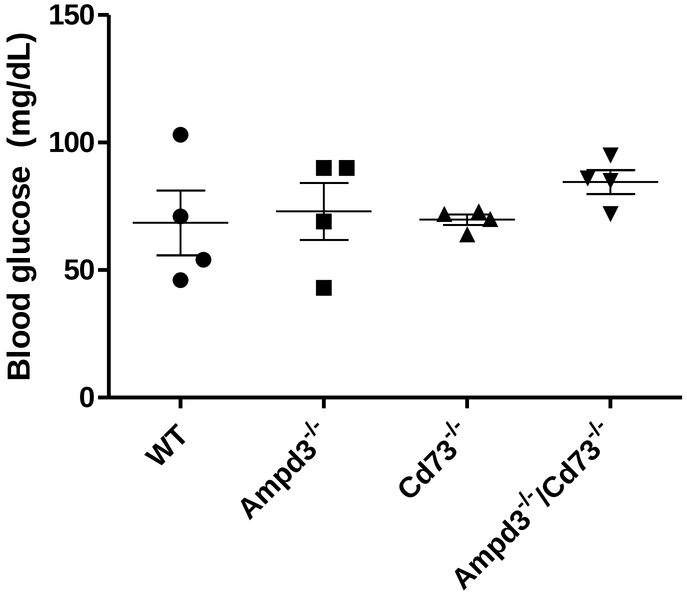

Figure S2. Fasting blood glucose levels. Mice from the four genotypes (n=4) were fasted overnight and blood obtained by tail clipping. Blood glucose was measured using a TrueTrack meter (Nipro Diagnostics, FL, USA), following manufacturer’s instructions.

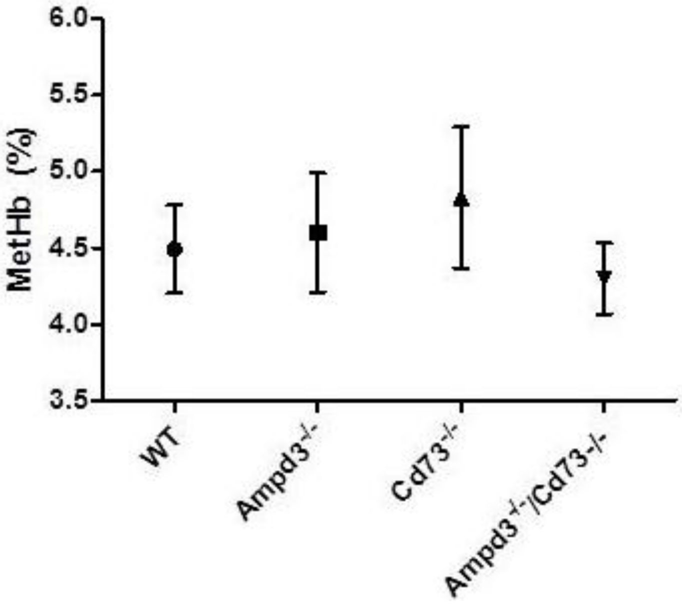

Figure S3. Methemoglobin (MetHb) levels in four genotypes, (n=4). The erythrocyte metHb levels are comparable among the four genotypes.

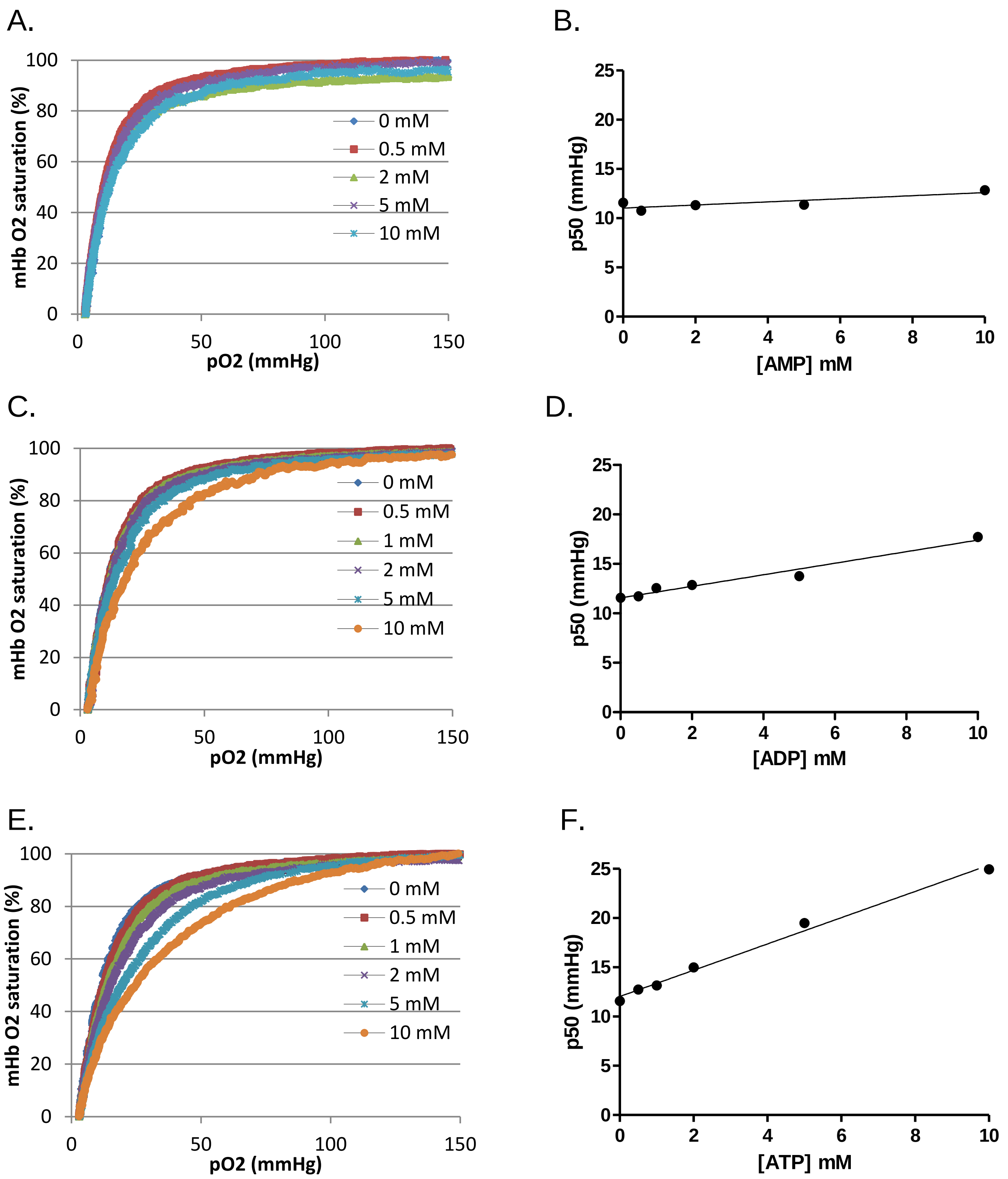

Figure S4. Adenine nucleotides' effects on p50 of mouse hemoglobin. Adenine nucleotide titrations of mouse hemoglobin p50: A and B. 5'-AMP ($m=0.16 \pm 0.05$, $r^2=0.73$); C and D. ADP, ($m=0.58 \pm 0.05$, $r^2=0.97$) and E and F. ATP ($m=1.33 \pm 0.06$, r^2-$=0.99$).

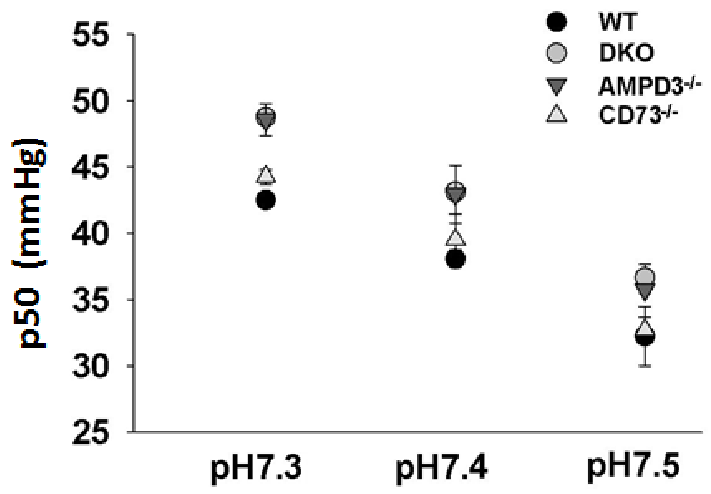

Figure S5. Change in Hemox buffer pH and p50 values of mouse erythrocytes of the four genotypes [n=2, DKO (double knockout): *AMPD3*$^{-/-}$/*Cd73*$^{-/-}$].

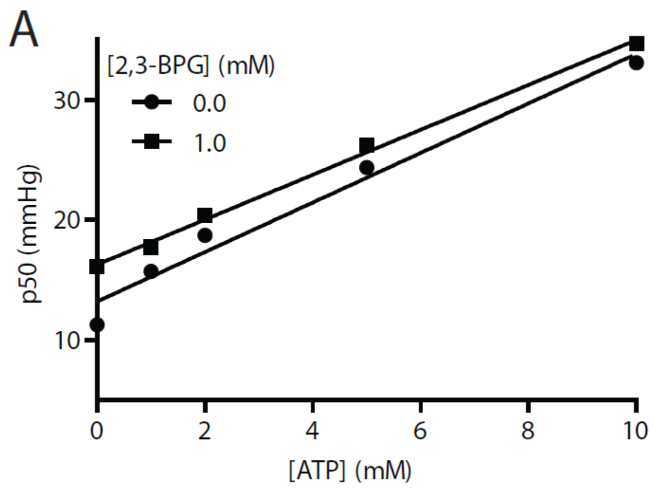

Figure S6, ATP's efficacy on Hemoglobin p50 is unchanged by addition of 2,3-BPG. ATP titration against human hemoglobin p50 in the presence and absence of 1mM 2,3-BPG.
